# A qualitative investigation of organizational challenges and facilitators to screening individuals experiencing homelessness for hepatitis C virus (HCV) in Houston, Texas

**DOI:** 10.1371/journal.pone.0273302

**Published:** 2022-08-22

**Authors:** Alane Celeste-Villalvir, J. Michael Wilkerson, Christine Markham, Lourdes Rodriguez, Vanessa Schick

**Affiliations:** 1 The University of Texas Health Science Center at Houston (UTHealth), School of Public Health, Division of Management, Policy and Community Health, Houston, Texas, United States of America; 2 The University of Texas Health Science Center at Houston (UTHealth), School of Public Health, Division of Health Promotion and Behavioral Sciences, Houston, Texas, United States of America; 3 The University of Texas Health Science Center at Houston (UTHealth), Center for Health Promotion and Prevention Research, Houston, Texas, United States of America; 4 St. David’s Foundation, Austin, Texas, United States of America; GITAM College of Pharmacy: GITAM Institute of Pharmacy, INDIA

## Abstract

**Background:**

Individuals experiencing homelessness may be at a disproportionately high risk for hepatitis C (HCV) because they may be more likely to engage in HCV risk behaviors. Community organizations that provide services to these vulnerable individuals can effectively screen, diagnose, and navigate them into HCV care. However, screening people experiencing homelessness for HCV at such organizations is limited by various challenges that remain understudied, including budgetary considerations and strategies to improve teamwork and communication. Accordingly, this study investigated the organizational challenges and facilitators to HCV screening of individuals experiencing homelessness as reported by homeless services providers.

**Methods:**

Staff (N = 21) at two community organizations in Houston, Texas, completed an interviewer-administered survey and a semi-structured interview in August 2020 to assess the challenges and facilitators to screening people experiencing homelessness for HCV. Interviews were coded, and a thematic analysis was conducted to identify challenges as well as facilitators to HCV screening among individuals experiencing homelessness.

**Results:**

Almost half of participants were employed in social services (42.86%; n = 9), while the remainder were employed in management/administration and health services. Barriers to HCV screening included funding, logistics, and resource-related challenges; and limited communication and collaboration around HCV screening. Facilitators to HCV screening included providing HCV education and training for all staff; and incentivizing, formalizing, and funding HCV screening.

**Conclusions:**

Community organizations can help minimize barriers to HCV screening among individuals experiencing homelessness by providing staff with training specific to HCV, client education around HCV and the screening process, and providing clients with incentives for participation, as well as by maximizing community and clinic partnerships to provide linkage to care and services to this high-risk population.

## Introduction

Hepatitis C virus (HCV), the most common bloodborne illness in the U.S., is a leading cause of death, and it costs the U.S. healthcare system billions each year [1,2]. Over 2 million people in the U.S. (about 1% of the U.S. adult population) are living with HCV, and more than 40% of people with chronic HCV are unaware of their status [3,4]. More than 50% of the HCV cases in the U.S. are concentrated in 9 states, including Texas [5,6]. Historically, the highest prevalence rates of chronic HCV in the U.S. were reported among “baby boomers” (individuals born between 1945–1965), but in 2018, the largest proportion of reported chronic HCV cases were among individuals aged 20–39 and 50–69 (the latter group are baby boomers) [7]. Furthermore, studies indicate that HCV prevalence among adults with experiences of homelessness ranges from 9.8% to 52.5% [8].

Screening and knowing one’s HCV status is the first step in the HCV care continuum, but only 56% of individuals with HCV are aware of their status [5,8,9]. For example, a study among 246 women experiencing homelessness in San Francisco found that approximately one-third of the women who were positive for HCV were unaware of their status [10]. Though previous screening guidelines focused on screening baby boomers, new Centers for Disease Control and Prevention (CDC) screening guidelines recommend screening all individuals 18 years and older at least once in their lifetime, all pregnant women during each pregnancy, and all individuals with risk factors for HCV (with repeat testing while risk behaviors continue) [5]. Being unaware of one’s HCV status not only increases the risk of infecting others, but it may also lead to serious health complications [7].

A lack of health insurance, disjointed medical care, and low health literacy have been cited as challenges to implementing HIV and HCV prevention programs among individuals engaged in substance use and individuals experiencing homelessness [8,11,12]. Researchers have found that individuals engaged in substance use receive less referrals to HCV specialists due to a lack of knowledge and skill-building around HCV among providers [8]. Researchers have also found that participants referred to another site for HCV-related care seldom show up [8,13]. However, strategies such as reflex testing (e.g., HCV antibody test first, then confirmatory HCV test, if antibody test is positive) may reduce the number of individuals who do not receive confirmatory testing following their initial screening [8,14]. Also, point-of-care testing such as rapid HCV tests, along with other same-day services (e.g., for medication), are effective ways to eliminate barriers for individuals experiencing homelessness, who often lack access to transportation [15].

Ending the HCV epidemic requires expanding the type of providers who screen for, diagnose, and treat HCV to include those in non-traditional, community settings [16,17]. Community organizations can effectively screen, diagnose, navigate high-risk individuals into care, and retain them in HCV care [18]. Screening for HCV in community settings can also help reach individuals who are disengaged from medical care in a way that is relevant to their circumstances [16,19]. Alternative settings for HCV screening have helped improve community awareness and identify individuals who were unaware of their HCV infection or aware of their HCV infection but not connected to treatment [17].

HCV screening is not a main priority for many individuals experiencing homelessness [15], who are at disproportionately high-risk for HCV because they are more likely to engage in known risk behaviors for HCV [20,21]. HCV is transmitted through contact with infected blood, most commonly through injection drug use [7,13,22,23]. Organizations providing homeless services may benefit from integrating healthcare for HCV and providing integrated services [20]. Integration of services for high-risk groups, such as individuals experiencing homelessness and those engaged in substance use, increase uptake of both screening and treatment for HCV [13]. However, it is critical to understand participant needs and characteristics to effectively integrate HCV care with other services for high-risk groups [13].

Multidisciplinary program models are essential to the prevention of HCV and HIV among high-risk groups [11]. Integrated HIV/HCV testing at a methadone clinic resulted in a testing rate of 79%, and HIV/HCV screening programs integrated to medication assisted treatment (MAT) programs found screening rates between 95–99% [13]. Providing screening, education, counseling, and case management at methadone clinics was shown to be an effective method of treating HCV and ensuring adherence to care [24]. Screening utilizing both rapid HCV and HIV tests at substance abuse treatment programs was also shown to be cost effective [25]. Rapid HCV tests offered in an MAT facility significantly increased HCV screening rates from 76% to 94% [13]. Integrated STI and HCV testing also led to an HCV testing rate of 92% among people who inject drugs [13]. A nurse-led HCV care model found that integrated programs promoted both testing and treatment initiation, with a 78% screening rate and a 100% cure rate for those who were positive for HCV [15].

Despite their great promise for improving HCV screening, integrated programs face challenges such as staff opposition to workload and training, as well as limited organizational capacity and resources [13]. Providing referrals to additional care is another challenge in less integrated programs (such as those that only provide screening) [13].

There are multiple gaps in the current literature related to integrated program models. First, studies on integrated models with HCV provide a quality of evidence that is low to moderate, with very few rigorous designs [13]. Second, HCV integration approaches varied across studies, making a comparison between studies and replication of program models difficult [13]. Third, studies on integrated models with HCV utilized small samples, and were implemented in settings that may not be generalizable to other locations and populations [15]. Fourth, there is scant information about strategies to improve teamwork and communication among teams implementing integrated HCV programs, although these strategies may be critical to the expansion of these programs and also increase retention [11]. Lastly, there is a limited understanding of budgetary concerns and considerations, which are critical to the implementation of integrated HCV program models [11].

In the present study, we investigated organizational challenges and facilitators associated with screening individuals experiencing homelessness for HCV. This study aimed to identify challenges and facilitators in screening individuals experiencing homelessness for HCV as reported by homeless service providers.

## Methods

### Study setting

This study was conducted at two community-based organizations that provide services to individuals experiencing homelessness in Houston, Texas, during August 2020. Site 1 is a small nonprofit organization (20 employees or less) with a mission to provide a faith-based community to rebuild the lives of people experiencing homelessness. This organization provides a mailing address and mail services, clothing and hygiene products, and hot meals. The clinic, a satellite community clinic part of the county hospital system, is integrated and co-located at the non-profit social services organization, providing health services onsite as well as referrals. Site 2 is a medium-sized nonprofit organization (49 employees or less) with a mission to provide essential services and coordination to help individuals move out of homelessness. This organization provides services such as laundry, showers, hot lunches, legal services, mail service, housing assessments, and case management. The co-located clinic, a satellite of a nationwide network of homeless clinics, is integrated and co-located at the nonprofit social service organization, providing health services onsite as well as referrals.

HCV prevalence in Texas is 23% higher than that in the U.S. [26]. It is estimated that more than 25% of Texans are at risk for HCV, and over 584,196 Texans have chronic HCV [27]. Since 1990, mortality from HCV increased by 71% in men and 29% in women in Texas, with the highest rates among Black/African American (2.8%) and Hispanic/Latinx (2.0%) individuals [27,28]. The highest proportion of HCV cases in Texas are located in (or near) major cities, such as Houston [27]. Harris County, where Houston is located, is the county with the largest population in Texas [26]. In Houston, individuals experiencing homelessness and engaged in substance use have been found to be twice as likely as non-homeless persons with no history of substance use to be positive for HCV [8].

### Study design

This qualitative study was conducted over several weeks in August 2020 with a convenience sample of 21 individuals employed at two community-based organizations in Houston, Texas. Participating organizations provide both health services (e.g., through nurses) and social services (e.g., through social workers) to individuals experiencing homelessness, including meals, clothing, showers, and laundry. These organizations provide HCV screening through a co-located Federally Qualified Health Center (FQHC).

Inclusion criteria for this study included being aged 18 years and older, being fluent in English, and having been employed at the organization for at least 6 months at the time of the study. Participating individuals received $20.

### Data collection

Participants completed a brief demographic survey prior to completing the semi-structured interview. The demographic data were collected via Qualtrics using an encrypted and password-protected tablet. The interviewer-administered demographic surveys took about 5 minutes to complete. Semi-structured interviews were audio-recorded and took between 45 minutes to 1 hour to complete. All participants provided informed verbal consent. The pseudonymized surveys and interviews were confidential, and no personal or identifying information was collected. Data were collected until saturation was reached and no new overarching themes or sub-topics emerged in the data [29]. The semi-structured interview guide included questions about participants’ experiences working with individuals experiencing homelessness, the nature of their job/role, their day-to-day activities, and their knowledge of HCV screening at their organization. Specifically, the interview focused on identifying challenges in providing screening for HCV and ideas that can help improve HCV screening.

### Data analysis

Survey data were cleaned on Microsoft Excel and analyzed with IBM SPSS Statistics Version 25 [30,31]. These data are primarily represented as measures of frequency (counts and percentages) and measures of central tendency (means and medians).

Interviews were audio-recorded, transcribed verbatim by a professional transcription company, and uploaded to ATLAS.ti 8 for content analysis [32,33]. A subset of interview transcripts was coded for distinct themes based on the meaning of participants’ words and phrases [32]. Preliminary codes were compared, and then examined for frequency, strength, and relationships in order to determine final coding taxonomy. A codebook was developed based on this preliminary coding process, and used to code the remaining transcripts. Following the coding of transcripts, an analysis of the consistent threads and ideas resulted in the identification of themes. This thematic analysis provided an overview of barriers and facilitators discussed by participants.

This study was approved by The University of Texas Health Science Center at Houston (UTHealth) Committee for the Protection of Human Subjects (IRB).

## Results

### Participant characteristics

A total of 21 employees of two community-based organizations that provide social and health services to individuals experiencing homelessness in Houston, Texas, participated in this study. Mean participant age was 41 years old (median: 38; SD: 12.83), with a range from 24 to 67 years old. Most participants were female (71%; n = 15) and identified as Black or African American (57%; n = 12) (Table 1). Participants had various levels of educational attainment: some college, Associate’s Degree, or technical degree (38%; n = 8); a Bachelor’s degree (24%; n = 5); and postgraduate studies (29%; n = 6).

**Table 1:** Demographic characteristics of study sample.

	(%)	(n)
Sample Distribution (by site)		
Site 1	62%	13
Site 2	38%	8
Gender		
Female	71%	15
Male	29%	6
Racial/Ethnic Identity		
Asian	5%	1
Black or African American	57%	12
Hispanic/Latinx, or Spanish	14%	3
White	14%	3
More than one racial/ethnic identity	10%	2
Education		
Grades 12 or GED	9%	2
Some college, Associate’s Degree, or Technical Degree	38%	8
Bachelor’s Degree	24%	5
Any post graduate studies	29%	6
Length of Time Employed at Organization		
6 months- 1 year	14%	3
1 year- less than 3 years	33%	7
3 years- less than 5 years	0%	0
5 years- less than 10 years	29%	6
More than 10 years	24%	5
Job Category		
Administration/Management (Program directors, managers, executive leadership)	29%	6
Social Services (case management, navigators, social workers, CHWs)	42%	9
Clinical Care (nurses, nurse practitioners, licensed vocational nurses, patient care technicians)	29%	6

Most participants had been employed at their respective organization between one year, but less than three years (33%; n = 7), and some had been employed for more than 10 years (24%; n = 5). Almost half of participants were employed in social services (42%; n = 9) as case managers, navigators, social workers, and community health workers (CHWs). The remaining participants were employed in management/administration (29%; n = 6) and in clinical roles (29%; n = 6).

### Thematic analysis

Themes that emerged from the transcripts fell into two categories: HCV screening barriers and HCV screening facilitators. Under the theme of HCV screening barriers, the prominent sub-themes were (a) funding, logistics, and resource-related challenges, as well as (b) limited information and collaboration around HCV screening. Under the theme of HCV screening facilitators, the prominent sub-themes were (a) providing HCV education and training to all staff and (b) the need to incentivize, formalize, and fund HCV screening.

Quotes include participant numbers and their role within the organization (e.g., social services). Individuals categorized in a “clinical” role are directly involved with the delivery and coordination of health services, whether in-house or by way of referrals.

#### Barrier: Funding, logistics, and resource-related challenges

*Funding*. Participants explained how grant-funded programs and their related reports and metrics determine which screenings are prioritized at their organization. HIV and TB were identified as more pressing needs than HCV because these grant-funded programs were tied to both required metrics as well as the needs of local shelters. Participant 11 (Female, Social Services) shared the following, “…HIV, they [the organization] push it because of the grant. But the TB skin test is because [of] the shelters, they [clients] have to have it for the safety of others.” In spite of the similarities between HIV and HCV (both bloodborne, infectious diseases), HCV was neither a grant-funded screening program nor a requirement of local shelters, and it was therefore not a top priority. Funding priorities thus determined what screenings are prioritized by organizations, and created shifts in employee roles in order to fulfill those prioritized organizational needs.

*Logistical and resource-related challenges*. The logistical and resource-related challenges at each organization provided additional barriers to HCV screening at both the client and organizational level. The steps required for participants to get the care they need were significant challenges for clients, and these challenges were recognized among staff at both organizations. Not having on-site screening, challenges with making appointments, lack of client access to phones, lack of client access to transportation, and long wait times are some of the resource-related and logistical challenges that make it difficult for clients to get HCV screening and other healthcare.

At one of the participating organizations, HCV screening was not provided on-site, and clients who wanted to get screened needed to go elsewhere to be tested. When asked about what the ideal HCV screening process would be in comparison to what it was at the time of the interview, Participant 4 (Female, Clinical) stated, “Having on-site testing, like immediately where it’s not putting it on the patient to have the possibility of going to get tested, it’s on us as an organization to get them tested.”

Staff at the second participating organization said that the process of trying to make an appointment or even showing up to their partner clinic with an appointment makes it difficult for clients to get the health services they need. As Participant 19 (Female, Admin/Management) stated,

…To get an appointment, you might have to sit on hold for 45 minutes, an hour to get an appointment. That’s not doable for most of our folks…. If they have a phone, they might have a government phone that’s got 200 minutes a month, you can’t use half of those in one phone call to make an appointment that’s three weeks away….

If a client is able to secure an appointment, they often face additional challenges with traveling to the appointment or being able to wait to be seen by a doctor. As Participant 19 further stated, “… the clinic makes themselves available for walk-in appointments, but a client might have to wait three, four, six hours.”

These logistical and resource-related challenges at the organizational level exacerbate individual-level challenges faced by clients, making it difficult for them to access HCV screening and other necessary healthcare.

#### Barrier: Limited information and collaboration around HCV screening

*Limited information*. Participants had limited information about screening eligibility as well as the steps and procedures associated with HCV screening. This was the case even among those whose role is directly related to health services. In fact, when asked about their familiarity with the criteria for HCV screening at their organization, the vast majority of participants were not familiar with these criteria (85.7%; n = 18). The few participants who had some familiarity with their organization’s HCV screening eligibility criteria (14.3%; n = 3) were individuals in clinical roles and who had been at the organizations for 5 or more years. However, these participants described previous screening guidelines, which exclude some of the more high-risk individuals such as individuals experiencing homelessness.

In addition to this limited information, staff also expressed that discussions around HCV were uncommon and that topics concerning the human body (such as HCV) were avoided, as they were deemed taboo. The lack of information and internal discussions about HCV are a barrier to HCV screening because it obscures the importance of HCV screening within the day-to-day operations of organizations.

Employees repeatedly shared that they were unsure of HCV screening eligibility and procedures because they were focused on the duties related to their own roles and left the clinical care to the co-located clinics and their staff. However, even clinic staff expressed lack of knowledge about HCV screening at the organizational level, suggesting that information deficits were not limited to staff whose roles were indirectly related or unrelated to HCV screening. At both organizations, it was common practice to refer clients asking about health services and screenings directly to the clinic without additional guidance and information provided, likely because of limited information sharing between the organizations. Participant 10 (Female, Admin/Management) stated, “…If they come in and ask that they want to get screened… I refer them to the clinic and they’re the ones that ask the screening questions.”

Most participants were unable to recall any internal discussions about HCV, and some stated flatly that no such conversations have happened in the time that they have been employed at their organization. Participants were also unable to recall seeing HCV in organizational materials or discussed in meetings, workshops, or trainings. Participant 18 (Female, Social Services) stated, “I just don’t hear it, I don’t know. I don’t hear it.”

The lack of discussion around HCV, according to one participant, was the result of discomfort in discussing topics relevant to HCV, even though such stigma and discomfort does not exist within the organization in reference to HIV. Participant 21 (Female, Social Services) revealed that the topic of HCV and anything “dealing with our bodies” is not discussed, and is considered both personal and taboo. She stated, “A knowledge gap, I would say, yes… I guess due to it being like a personal… taboo issue…” Anything dealing with the human body and anything that one “can be judged for” is not often discussed openly among staff.

*Limited collaboration*. Concerns around collaboration and information sharing between teams and between organizations and their clinic partners were identified as a barrier to HCV screening, as it hindered the knowledge of non-clinical staff and the promotion HCV screening. The lack of collaboration and information sharing creates teams focused on their own jobs and unfamiliar with what other teams are doing. Formalizing partnerships between social service organizations and co-located clinic partners, along with sharing information about what services are offered at each organization, can help all staff be informed about services available to clients.

When describing the navigation process between the two organizations, Participant 16 (Female, Social Services) shared, “So there’s no black and white… there’s nothing in papers, nothing written down, there’s no procedure. I usually just go to Google.” Education, formal partnerships, and sharing information were commonly mentioned as strategies to improve collaboration and promote health services and screenings. Some of the barriers to HCV screening are the result of limited communication, information, and collaboration between partners, and limitations in coordinated linking protocols between the clinics and their partner social service providers.

#### Facilitator: HCV education and training for all staff

When discussing ways to improve and promote HCV screening, most participants focused on providing training and education to staff. Specifically, they mentioned that education and training around HCV and the HCV screening process should be available for all staff, including non-clinical staff, who are often the first to come face-to-face with clients. Not having the necessary knowledge and education about HCV discouraged staff from engaging with clients around this subject, as they feared earning a reputation among clients as being misinformed.

Educating and training staff was seen as critical to increasing confidence in providing clients with information. Having adequate training and education about HCV and knowledge about the organization’s screening eligibility and process increases staff confidence in providing guidance to clients. Participant 8 (Male, Social Services) shared, “I haven’t had any training in hepatitis C. … if we don’t understand the importance of it or understand the background of it, how can we teach the client?” Additional training and education suggestions to facilitate HCV screening included having posters around the organization and the clinic, webinars and seminars, and having an educational DVD playing in the office.

With adequate HCV education and training available to all staff at the organizations, clients may be able to receive HCV screening information from staff who are informed and confident in their knowledge.

#### Facilitator: Incentivize, formalize, and fund HCV screening

Current HCV screening efforts at each organization were underfunded, informal, and excluded incentives for clients. When discussing ways to facilitate HCV screening, participants mentioned that HCV screening should be incentivized, formalized, and funded in order to become as much of a priority as other screening programs within their organizations. Incentives at the client level were identified as effective means to promote programs and encourage participation from clients. Having homeless health services (including HCV screening) tied to a grant-funded program was mentioned as an organizational-level method for prioritizing screening. In addition, having set standards and protocols around HCV screening was uplifted as an organizational-level strategy to keep HCV a priority among staff.

Having organizational HCV screening efforts funded and tied to grant programs was also mentioned as a strategy to prioritize HCV screening at the organizational level. Health screenings tied to grant programs were identified as those that are emphasized and regularly discussed among staff and with clients. Having to meet grant-related metrics and objectives serves as an incentive to keep HCV screening as an organizational priority. As Participant 11 (Female, Social Services) stated,

Maybe we can get it to where it can become a grant, that will help. And then they’ll [other staff] push it…. Right now, they were talking about a high blood pressure grant that they got so they’re recruiting patients for the high blood pressure test… But we have to have the grant in order for facilities to work.

Having formal standards and protocols around HCV screening was also highlighted several times as a potential way that HCV can be prioritized and HCV screening encouraged at the organizational level. Participant 17 (Female, Clinical) said that,

I think the organization need to have the protocols. So, if there is a standard protocol, then we have to test patients. So and so age and, this length like intervals. If we have those standard protocols, then we will do it. I don’t see any policy, about the hepatitis C mandatory testing…

Establishing formal standards and protocols around HCV screening, funding HCV screening efforts, and client-level incentives can help improve HCV screening and elevate HCV as a priority among staff when interacting with clients.

## Discussion

The present study investigated the organizational challenges and facilitators to providing HCV screening to individuals experiencing homelessness from the perspective of homeless services providers. Participants identified (a) funding, logistics, and resource-related challenges, as well as (b) limited information and collaboration around HCV screening as the primary barriers to providing HCV screening to individuals experiencing homelessness. Screening facilitations included (a) providing HCV education and training to all staff and (b) the need to incentivize, formalize, and fund HCV screening.

Other studies on HCV have noted that there is a limited understanding of budgetary concerns and considerations, which are important to the adequate implementation of integrated program models [11]. In the case of HCV screening at the two participating organizations, established grant-funded priorities set the agenda for which screenings are prioritized. Policymakers and funders can move the needle on HCV screening by increasing funding opportunities available to community-based organizations and by making HCV screening as much of a funding priority as other infectious diseases known to disproportionately impact individuals experiencing homelessness. Organizations can use these resources to provide staff training and education, increase staffing, and eliminate existing procedural and structural barriers hindering access to screening.

In addition to grant-funded priorities, several logistical and resource-related limitations were identified as barriers to screening individuals experiencing homelessness for HCV, including not providing on-site screening and rapid testing. Previous studies have reported that high-risk individuals who are referred out for HCV-related care seldom show up for appointments at another location due to challenges related to transportation [8,13]. Having integrated HCV screening would allow individuals to have convenient access to a one-stop shop of services, which has been shown to increase uptake in HCV screening [13]. Researchers have concluded that point-of-care testing strategies (such as rapid testing) and same-day services eliminate barriers including long wait times and ongoing transportation needs [8,13,15]. Understanding the needs and priorities of high-risk groups can support the effective implementation of integrated HCV care with other social and health services [13].

Another substantial challenge around HCV screening is limited information and collaboration, as well as limited knowledge HCV and discomfort discussing HCV. In fact, HCV screening and HCV-related care coordination is not mentioned in either of the participating organization’s websites, though one organization’s website mentions HIV and TB testing. Previous studies have found that individuals engaged in substance use receive less HCV care referrals, a challenge that can be alleviated through provider education about HCV care, stigma, and bias against people engaged in substance use [8]. This gap in knowledge presents organizations with an opportunity to provide staff with training about HCV, HCV screening recommendations, and HCV screening process. Posting easily visible and accessible materials around the facility can also help increase HCV education among staff.

Issues around collaboration and information sharing were also identified as challenges to screening individuals experiencing homelessness for HCV. Previous studies have reported challenges in providing referrals, and this breakdown in collaboration and information sharing is a significant challenge to providing information, education, and access to HCV screening to individuals experiencing homelessness [13]. Because clients at both the social services organizations and their neighboring clinic partners overlap, a commitment to innovative, full integration can prove highly effective in increasing HCV screening and treatment, as researchers have shown in past studies [16,17]. Future studies should consider exploring the wide spectrum and variability of integration approaches within community-based organizations.

The incentivization, formalization, and funding of HCV screening were mentioned by participants as additional ways to facilitate HCV screening. Establishing standard and formalized protocols for HCV screening (e.g., frequency and eligibility of screening) can help mobilize staff to screen more individuals and elevate HCV as an organizational priority. Clinical staff would benefit from education about the most recent HCV screening guidelines set forth by the CDC and other health agencies. Social service and administrative staff would benefit from knowledge around their clinic partner’s procedures for HCV screening in order to inform clients of what to expect and how to prepare for HCV screening. Another strategy that can help prioritize HCV screening is having screening tied to grant funding, which will require staff to meet reporting metrics and other grant-related goals.

The present study had several limitations. As is common with qualitative studies, the transferability of this study may be limited and its findings may not be applicable to other organizations, geographic locations, and populations. The self-reported and cross-sectional data only have the potential to provide a point-in-time, snapshot estimate of the individuals interviewed. This study was conducted during the COVID-19 pandemic, during which programs were operating at minimized capacity to adhere with social distancing and other safety precautions. Because of the COVID-19 pandemic, the participants of this study were not representative of all the positions/roles within the organizations, as many employees at the organizations were working remotely. As a formative study, analyses were exploratory and intended to inform hypothesis generation. Further research is needed to confirm findings and to further understand the challenges and facilitators facing integrated health/social service organizations providing services to individuals experiencing homelessness.

In spite of the aforementioned limitations, the present study makes a unique contribution to the literature about HCV screening among individuals experiencing homelessness. This study provides insight into the budgetary concerns and considerations critical to implementing integrated program models, a known gap in the literature [11]. This study also provides some insight into specific teamwork and communication challenges. The lack of knowledge of strategies for improving these challenges has also been identified as a gap in the current body of literature and is critical to the expansion of integrated programs [11]. To the best of our knowledge, universal HCV screening among those with experiences of homelessness is not standard practice, and no known studies have examined why this is so and the challenges and facilitators associated with screening people experiencing homelessness for HCV. Future studies should consider piloting and evaluating the impact of universal HCV screening within integrated programs serving people experiencing homelessness.

Qualitative research concerning the organizational challenges and facilitators to screening individuals who are homeless for HCV is scarce. Much of the literature concerning HCV is focused on quantifying the burden of disease and rates of infection, treatment, adherence, and screening rates (e.g., Cole et al., 2019; Weick, Watson, & Salgia, 2019; Fuster & Gelberg, 2019) [21,34,35]. The day-to-day context and experiences of individuals employed at community-based organizations who are on the frontlines providing homeless services is missing from the scientific literature, and may provide meaningful insight that can improve how HCV education and screening is provided to high-risk individuals.

## Supporting information

S1 File (XLSX)
